# Linguistic feature of anorexia nervosa: a prospective case–control pilot study

**DOI:** 10.1007/s40519-021-01273-7

**Published:** 2021-07-26

**Authors:** Vittoria Cuteri, Giulia Minori, Gloria Gagliardi, Fabio Tamburini, Elisabetta Malaspina, Paola Gualandi, Francesca Rossi, Milena Moscano, Valentina Francia, Antonia Parmeggiani

**Affiliations:** 1 Regional Center of Feeding and Eating Disorders in developmental age, Child Neurology and Psychiatry Unit, IRCCS Istituto delle Scienze Neurologiche di Bologna, Bologna, Italy; 2 Department of Medical and Surgical Sciences, University of Bologna, Bologna, Italy; 3 Department of Classical Philology and Italian Studies, University of Bologna, Bologna, Italy

**Keywords:** Anorexia nervosa, Clinical linguistics, Linguistic marker, Eating Disorders, Adolescence

## Abstract

**Purpose:**

Attention has recently been paid to Clinical Linguistics for the detection and support of clinical conditions. Many works have been published on the “linguistic profile” of various clinical populations, but very few papers have been devoted to linguistic changes in patients with eating disorders. Patients with Anorexia Nervosa (AN) share similar psychological features such as disturbances in self-perceived body image, inflexible and obsessive thinking and anxious or depressive traits. We hypothesize that these characteristics can result in altered linguistic patterns and be detected using the Natural Language Processing tools.

**Methods:**

We enrolled 51 young participants from December 2019 to February 2020 (age range: 14–18): 17 girls with a clinical diagnosis of AN, and 34 normal-weighted peers, matched by gender, age and educational level. Participants in each group were asked to produce three written texts (around 10–15 lines long). A rich set of linguistic features was extracted from the text samples and the statistical significance in pinpointing the pathological process was measured.

**Results:**

Comparison between the two groups showed several linguistics indexes as statistically significant, with syntactic reduction as the most relevant trait of AN productions. In particular, the following features emerge as statistically significant in distinguishing AN girls and their normal-weighted peers: the length of the sentences, the complexity of the noun phrase, and the global syntactic complexity. This peculiar pattern of linguistic erosion may be due to the severe metabolic impairment also affecting the central nervous system in AN.

**Conclusion:**

These preliminary data showed the existence of linguistic parameters as probable linguistic markers of AN. However, the analysis of a bigger cohort, still ongoing, is needed to consolidate this assumption.

**Level of evidence III:**

Evidence obtained from case–control analytic studies.

**Supplementary Information:**

The online version contains supplementary material available at 10.1007/s40519-021-01273-7.

## Introduction

Over the last few years, a growing body of linguistic studies have been devoted to speech and language disorders: this fairly new branch of linguistics is called “Clinical Linguistics” [1] and it helps supporting speech and language therapists and neuropsychologists. Thanks to automated computational methods, progress in the field has been breathtaking. Sophisticated Natural Language Processing (NLP) techniques, newly developed, have been used to analyze written and spoken texts, revealing latent patterns and regularities in pathological speech.

These subtle language disruptions can be used as “digital biomarkers”, namely objective, quantifiable behavioral data which can be collected and measured by means of digital devices, allowing for a low-cost pathology detection and classification.

Within this context, a number of works have been published on the “linguistic profile” of various clinical populations [2–5]: linguistic deficits have been reported in several neurodegenerative diseases such as dementia [6, 7], where language disruption is a common finding both in the earliest stages and in full-blown pathology; alterations have been extensively described in scientific literature on dysphonia and dysarthria, especially in the hypokinetic forms resulting from damage to the basal ganglia (such as in Huntington's disease, Progressive Supranuclear Palsy or Parkinsonism) [8–11]. Some studies deal with the linguistic habits of psychopathologies, e.g., schizophrenia [12–14], personality disorder [15], anxiety and depression [16–22]. However, a very limited number of papers have been devoted to linguistic changes in patients with eating disorders [23–29].

Within the large field of eating disorders, Anorexia Nervosa (AN) has drawn increased interest from the linguistic community in the last few decades.

The complexity of this disorder depends on the almost constant presence of psychiatric comorbidity and medical morbidity, as well as secondary problems associated with malnutrition. Indeed, AN is associated with cognitive and emotional disturbances [30], although it is not yet clear whether as a cause or a consequence of the disorder or malnutrition.

Scant information exists about the incidence and prevalence of AN due to the lack of representative epidemiologic data and different assessment methods, which limit the meaningfulness of statistical evidence [31]. Incidence rate could be underestimated on account of the fact that the majority of individuals experience the disease as egosyntonic and do not get used to treatment, so the majority of AN patients in the community do not enter the mental healthcare system [32].

All studies report higher incidence in women and girls than in men and boys, with gender ratios of approximately 10/1 to 15/1. The incidence of AN requiring inpatient treatment in Italy for the age group 10–19 years is 22.8 per 100.000 women and 2.0 per 100.000 men [33]. Good evidence supports the conclusion that the rate of first diagnosis of AN is highest among individuals of 15–20 years of age in both males and females [32].

From a psychological point of view, weight loss is often viewed by AN patients as a sign of extraordinary self-discipline and perfectionism, whereas weight gain is perceived as an unacceptable failure. Inflexible thinking is a core feature of the disorder, as well as rigid behavior, almost disconnected from the somatic experience, weak set shifting (reduced ability to move back and forth between tasks), weak central coherence (attention to details rather than to the general picture), a sensitivity to praise and reward and anxiety sensitivity or harm avoidance [34–36].

A prompt identification and treatment of symptoms are linked to better outcomes [37]. Unfortunately, as already pointed out, the diagnosis of AN is often elusive, and more than one half of all cases go undetected in the primary care setting [38]. Therefore, current research continues to emphasize the need for novel reliable strategies to identify even early warning signs.

To date, only few studies investigate speech in people with eating disorders [23–27]. These studies mostly focus on the differences characterizing the texts of self-presentation written by individuals who publicly defend AN as a lifestyle (“pro-ana”), rather than on the language uses of affected patients [23, 27].

## Aim of the study

The primary aim of this study is to better understand the psychopathological elements of AN aided by recent developments in clinical linguistics. Patients with AN share similar psychological features, like disturbances in self-perceived body image, inflexible and obsessive thinking and anxious or depressive traits [39–41]. We hypothesize that these characteristics can result in altered linguistic patterns (i.e., subtle anomalies in verbal production) and be detected using NLP tools. In particular, we believe that cognitive and emotional disturbances of AN can correlate with abnormalities in the written productions of the patients, at the syntactic, lexical, and semantic levels. Moreover, we assume that these slight language disruptions can be easily identified in the texts through NLP methods and employed as “digital linguistic biomarkers”.

In consideration of the widespread diffusion of AN among people of developmental age, the long-term aim of this pilot study is to be able to create an ecological tool (i.e., a psychometric instrument which is able to predict behaviors in real-world settings), potentially applicable both in the clinical and school contexts, which allows to support early detection and treatment of the disease. To the best of our knowledge, this is the first study on the linguistic profiling of AN-affected individuals in Italy.

## Materials and methods

This work is an observational prospective case–control study. The study began before SARS-COV2 pandemic and it is still ongoing, with full results expected in 2022. It was approved in December 2019 by the Metropolitan Bioethics Committee in Bologna with protocol number 683/2019/Oss/AOUBO. Study participants were divided into two groups: the Anorexia Nervosa group (ANG) and the Control Group (CG), with a ratio of 1:2. Fifty-one participants are currently enrolled; they are divided as follows:

- ANG: 17 patients with a clinical diagnosis of Anorexia Nervosa according to DSM-5 and Eating Disorder Inventory-3 (EDI-3) questionnaire [42], recruited at the Regional Center of Eating disorders in Bologna;
- CG: 34 high school students from Bologna matched by sex, age and educational level (school grade/type of secondary school) compared to ANG.

First, through a short self-reported questionnaire, we surveyed all participants to ascertain their language proficiency in Italian.

In fact, bilingualism and multilingualism are the norm rather than the exception in today's Italy. Our preliminary test aimed at assessing both quality and quantity of bi- or multi-lingual experience, to remove from the sample poor productions due to scarce exposure to standard Italian.

To ensure privacy, each participant was identified by a code rather than their personal name. Inclusions criteria are reported in Table 1.

**Table 1:** Inclusion criteria for participant enrollment

AN	CG
Age: 14–18 Diagnosis of Anorexia Nervosa (DSM-5, EDI-3) Fair level of communication skills in standard Italian (Language History Questionnaire) Written informed consent	Age: 14–18 BMI ≥ 18.5 Fair level of communication skills in standard Italian (Language History Questionnaire) Written informed consent

**Fig. 1:**
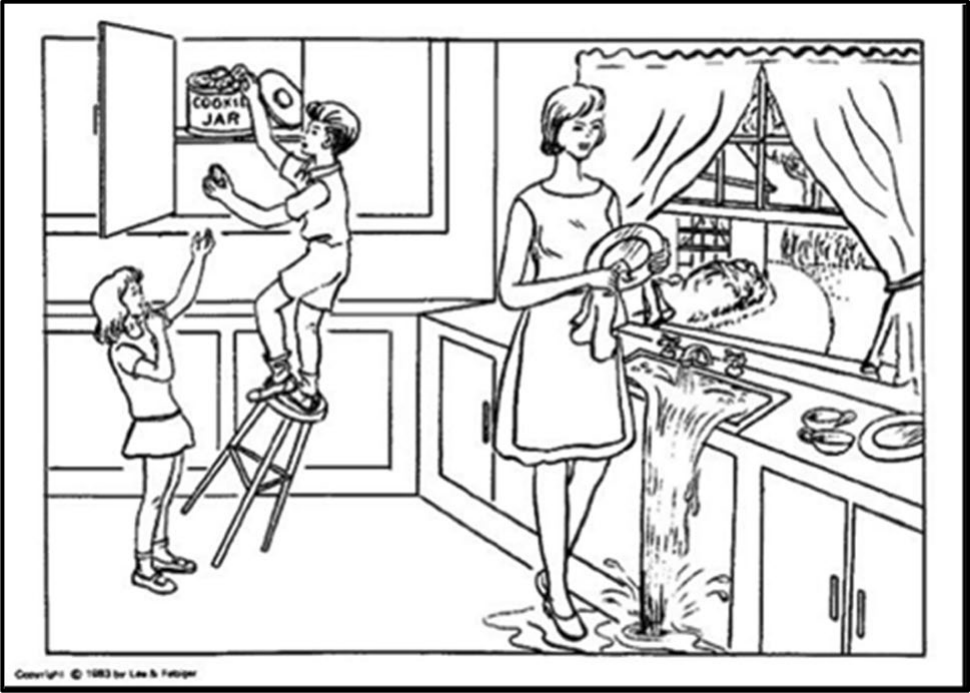
The Cookie theft

Second, all participants were asked to complete three “linguistic tasks”, namely to produce three short written texts (around 10–15 lines long), according to the literature [24–26, 29]:

1. Personal task: “How would you describe yourself? (Please, talk about your physical and personality traits, your hobbies, etc.)”.
2. Neutral task: “How do you usually spend time with your friends?”
3. Description of a complex picture: the black and white picture “Cookie theft” from the BDAE—Boston Diagnostic Aphasia Examination Battery [43] (Fig. 1).

These linguistic tasks were submitted to ANG as part of clinical activities and to the CG during ordinary school activities.

For ANG, we collected clinical data as follows: gender, age, BMI, age of onset and duration of illness, amenorrhea, purging behavior, pharmacological therapy, and comorbidity.

## Data analysis

Linguists manually digitized the handwritten texts. This corpus has been enriched by adding linguistic information at the lexical and morphosyntactic levels: all the sentences have been automatically analyzed by the Turin University Linguistic Environment—TULE [44], based on the TUT—Turin University TreeBank tagset, a collection of Italian sentences annotated at a morphosyntactic, syntactic and semantic level, with dependency-oriented representation format.

A multidimensional parameter analysis has been performed on the corpus: after examining the relevant literature, we selected a wide range of linguistic/stylometric indexes to be tested to determine their relevance in the discrimination between AN and control subjects. For a thorough description of the indexes, please refer to Beltrami and colleagues (2018) [7]. In addition, we used the software LIWC (Linguistic Inquiry and Word Count) [44, 45], a text analysis program, which counts the percentage of different lexical categories, to capture the participants’ social and psychological states (i.e., emotions, thinking styles, social concerns).

For all the linguistic variables, we evaluated differences of distribution between ANG and CG with the Kolmogorov–Smirnov non-parametric test. We choose a non-parametric approach due to the small sample size.

For ANG, we evaluated the clinical data mentioned above: sex, age, weight, height, BMI, onset and duration of illness, presence of purging behavior, amenorrhea, both physical and psychological comorbidity, pharmacological therapy with SSRI (e.g., fluoxetine, sertraline, fluvoxamine), anxiolytics (e.g., benzodiazepines) or antipsychotics (e.g., olanzapine, quetiapine).

We performed a statistical descriptive analysis for all clinical data. Spearman correlation index was calculated between linguistic and clinical data of ANG, to evaluate the correlation between changes in language use and disease severity.

## Results

Age and schooling differences of the enrolled participants (Table 2) are not statistically relevant (*p-value* > 0.05) based on the Kolmogorov–Smirnov test; thus, the sample is well balanced as regards each variable.

**Table 2:** Demographic characteristics of the sample

Group	N	Age (mean ± sd)	Years of education (mean ± sd)
ANG	17	16 ± 1.37	11.06 ± 1.34
CG	34	16 ± 1.35	11.15 ± 1.28

With regard to the clinical data, ANG was 100% female, with an average age of onset of the disease at 14.18 years (SD 1.54) and an average duration of the disease of 1.82 years (SD 1.29). Mean MBI was 17.04 (SD 1.56). Twelve patients (70.6%) were being treated with psychopharmacological therapy as follows: 41% (N 7) only with SSRI, 6% (N 1) only with antipsychotics, 24% (N 5) with a polypharmacotherapy.

In our AN sample, 58.8% (N 10) of patients were in secondary amenorrhea and 11.8% (N 2) were in primary amenorrhea; 29.4% (N 5) had purging behavior. In this group, we also observed comorbidity in 47.1% (N 8) of cases. The comorbidities observed were depressive disorder (N 6), anxiety disorder (N 6), and obsessive–compulsive disorder (N 2).

We calculated the number of words produced by each group for each task (Table 3). As corroborated by the statistical analysis, the three tasks show different “elicitation power” (Kruskal–Wallis non-parametric test with Dunn's multiple comparison. ANG: *chi-squared* = *8.1986, p-value* = *0.01658*; CG: *chi-squared* = *23.93, p-value* = *6.362e-06*). The “personal task” (task 1) prompted richer responses in both samples.

**Table 3:** Text length produced in the three tasks by ANG and CG

Task	ANG (mean ± sd)	CG (mean ± sd)
Task1 (Personal)	98.63 ± 42.94	105.5 ± 35.05
Task2 (Neutral)	61.53 ± 40.98	68.56 ± 31.55
Task 3 (Description of picture)	81.50 ± 40.02	77.15 ± 24.13
Overall	80.22 ± 43.16	83.74 ± 34.18

Given the large quantity of linguistic indexes that we analyzed, we report here only statistically relevant results. Findings are summarized in Table 4. Please refer to the supplementary materials for the full list of values assumed by the linguistic features in both groups.

**Table 4:** Results of the linguistic analysis

Linguistic variable	Task 1 (Personal)	Task 2 (Neutral)	Task 3 (Description of picture)	Overall
LEX_ContDens			D = 0.441 *p* = 0.024 *	
LEX_PoS_ADV	D = 0.412 *p* = 0.043 *			
LEX_PoS_CONJ				D = 0.235 *p* = 0.046 *
LEX_PDEIXIS		D = 0.412 *p* = 0.043 *		
LEX_HonoreR			D = 0.412 *p* = 0.038 *	D = 0.255 *p* = 0.022 *
SYN_NPLENSD		D = 0.411 *p* = 0.043 *		
SYN_GRAPHDISTM				D = 0.284 *p* = 0.008 **
SYN_SLENM	D = 0.412 *p* = 0.043 *			D = 0.284 *p* = 0.008 **
SYN_SLENSD	D = 0.412 *p* = 0.038 *			D = 0.245 *p* = 0.0337 *
LIWC_WPS		D = 0.412 *p* = 0.043 *		D = 0.245 *p* = 0.034 *
LIWC_SIXLTR			D = 0.441 *p* = 0.024 *	D = 0.333 *p* = 0.001***
LIWC_DIC		D = 0.441 *p* = 0.024 *	D = 0.588 *p* = 0.0008 ***	
LIWC_PERCP		D = 0.412 *p* = 0.043 *		
LIWC_PRES			D = 0.412 *p* = 0.043 *	

The significant p-value is indicated for the corresponding feature and task, with *p < 0.05; **p < 0.01; ***p < 0.001

The analyzed Lexical Indexes, showing statistically relevant differences between ANG and CG, are the following:

1. **Content Density (LEX_ContDens)**: ratio between open-class words like nouns, verbs, adjective and adverbs (lexical words) and closed-class words (grammatical words). ANG showed a higher content density in task 3 compared to CG.
2. **Part of Speech rate (LEX_PoS_ADV and LEX_PoS_CONJ**): ANG showed a lower frequency of conjunctions overall, and a lower frequency of adverbs in task 2 compared to CG.
3. **Personal deixis rate (LEX_PDEIXIS**): frequency of personal deictic expressions in written texts. ANG showed a lower use of personal deixis in task 2 compared to CG.
4. **Lexical richness (LEX_HonoreR)** [51]: proportion between words used only once and total number of words. ANG showed a higher lexical wealth in task 3 and overall compared to CG.

For Syntactic Indexes, differences between ANG and CG are indicated as follows:

1. **Number of dependent elements linked to the noun (SYN_NPLENSD)**: complexity of the noun phrase. ANG showed a lower complexity in task 2 compared to CG.
2. **Global Dependency Distance (SYN_GRAPHDISTM)**: syntactic complexity, quantified as the length of the arcs in the dependency tree. ANG showed a lower complexity overall, compared to CG.
3. **Utterance length (SYN_SLENM and SYN_SLENDS)**: length of the sentence, that is, average number of words per sentence. ANG showed a lower utterance length in task 1 and overall compared to CG.

Among indexes investigated using the software LIWC, differences between ANG and CG are indicated as follows:

1. **Words per sentences (LIWC_WPS)**: ANG showed a lower average of words per sentences in task 2 and overall compared to CG.
2. **>6-letter Words (LIWC_SIXLTR)**: ANG showed a higher count of >6-letter words in task 3 and overall compared to CG.
3. **Dictionary word count (LIWC_DIC**): ANG showed a lower percentage of words belonging to the LIWC dictionary in task 2 and overall compared to CG.
4. **Perceptual process (LIWC_PERCP)**: use of words referring to the senses (e.g., “see”, “hear”, “feel”). ANG showed a lower word count in task 2 compared to CG.
5. **Use of present tense (LIWC_PRES)**: ANG showed a lower use of the present tense in task 3 compared to CG.

Within ANG, results of correlation between clinical data and linguistic variables, calculated with Spearman, were not statistically significant. Additional data are needed to evaluate the correlation with disease severity.

## Discussion

The linguistic profiling of AN and other eating disorders remains to date mostly unexplored. Moreover, all studies published so far concern themselves with verbal production in a Germanic language, namely English, German or Norwegian. Given the peculiar typological (i.e., morphosyntactic) features of the Italian language, these results cannot be readily generalized and applied to Italian. Based on our knowledge, no other similar study has been conducted in Italy so far.

The first observation we can make by looking at the results regards the selection of the linguistic task: the most effective stimulus in distinguishing ANG and CG was the description of a complex picture. This finding is not surprising: according to Chung [46], linguistic tasks not directly pertaining to psychological and bodily states provide a non-reactive way to explore social and personality processes. However, in our opinion, aggregated tasks (“overall”) represent the best testing ground for the evaluation of subtle linguistic alterations. As a matter of fact, data scarcity is one of the major bottlenecks for Artificial Intelligence (AI) and NLP systems: in our study, the different tasks provide different contributions to describing the subject’s psychological state and their linguistic behavior; however, a larger amount of data allows a better characterization of verbal disruption in ANG.

Based on the current data, mostly focused on language in pro-anorexia blogs, the analysis of linguistic cues of emotional processes shows that pro-anorexics usually adopt more positive emotional words (e.g., “happy”, “good”), a lower rate of anxiety words (e.g., “afraid”, “scared”) and fewer cognitive mechanism words (specifically insight and causation words, e.g., “cause”, “realize”) than recovering anorexics [23, 27]. Moreover, pro-anorexics display lower levels of self-directed attention, since they make fewer first-person singular self-references; their texts contain more present-tense verbs and fewer past tense verbs, suggesting a focus on the present experience rather than on the past. Compared with recovery and control blogs, pro-eating disorder written productions contain a high proportion of exclamation marks but much fewer question marks, indicating a strong self-affirmation [47] and a reduced tendency to express insecurity and fears. This may also reflect a form of complexity reduction at the syntactical level [27]. The strong focus on oneself enters into combination with a low social relatedness. Pro-ana bloggers appear to be less connected with the outside world and real-life relationships [48]: this tendency is further supported by a low third-person plural pronoun use. Taken together, these observations are consistent with an interpretation of pro-anorexics’ language use as a coping strategy aimed at stabilizing them emotionally: these subjects experience a sense of control over the illness, and thus deploy a mechanism of self-defense.

In our sampling, syntactic reduction appears as the most relevant trait of ANG productions. In this respect, several indexes emerged as statistically significant (see Table 4). Distinguishing lexical features of our cohort include: Content Density, i.e., the ratio of open-class words to closed-class words, Lexical Richness calculated as R—Honoré’s statistic, rate of Adverbs, Conjunctions and personal deixis, incidence of LIWC2007 Dictionary (LIWC_DIC). At the semantic level, our data show a lower incidence of lexical units related to perceptual processes (LIWC_PERCP, i.e., multiple sensory and perceptual dimensions associated with the five senses) in AN patients with respect to controls. The most frequently described trait of AN, namely the lower use of first-person singular pronouns [23, 27] is not confirmed by our data, neither as the plural ones. The analysis of temporal focus is also controversial: in contrast with the work of Lyons et al. [23], written texts by CG contain more present-tense verbs (LIWC_PRES), which objects to the presumed attentional focus on the here-and-now of AN patients. Furthermore, none of the readability features turn out to be statistically relevant, except for the usage of long (> 6-letter) words (LIWC_SIXLTR), which recur more frequently in ANG.

Could this syntactic reduction be linked to the brain atrophy of AN patients? It is now well documented that patients with AN show brain modification (mostly reversible with refeeding) such as lower volumes of total brain (gray matter, white matter, cerebellum and insula, with higher volumes of cerebrospinal fluid and cerebral ventricles) [49]. This brain volume loss potentially leads to neuropsychological deficits, i.e., altered visuospatial functioning, reduced perceptual organization/reasoning and increase drive for thinness [50]. However, the clinical relevance of these cerebral alterations is currently poorly understood.

Two interesting studies conducted by Skårderud [25, 26] investigate the body’s symbolic role in the course of the illness. They call “concretized metaphors” the striking clinical feature of concreteness of symptoms, due to body image fluctuation, which means that there is a psychic equivalence between physical and psychic reality (e.g., ‘emptiness/fullness’, ‘purity’, ‘spatiality’, ‘heaviness/lightness’ ‘solidity’, ‘removal’). The ‘as-if’ quality of the more abstract meaning of the metaphor is lost, and the metaphor becomes instead an immediate concrete experience [25, 50]. These observations have been interpreted as evidence for the impairment of the psychological processes underlying the capacity to make mental representations. With this respect, semantics could represent a promising direction for future research.

In conclusion, the preliminary findings of our study—the first one considering Italian, a Romance language—suggest that it is possible to identify linguistic parameters as probable linguistic markers of AN. Since this study is still ongoing, it will be possible to collet additional data by increasing the number of ANG and CG cases to be examined. A larger cohort will make it possible to evaluate how such comorbidities as anxiety or depression, active often at a sub-clinical level, may also affect language as reported in the literature [17, 19, 21].

If these preliminary results are confirmed, the use of an automatic system (e.g., Machine Learning classifiers) analyzing and classifying patients’ language use in written productions may offer significant support for the identification of both overtly pathological and sub-clinical conditions. Compared to the clinical instruments currently available, linguistic analysis is an ecological, low-cost and non-invasive method that can also be administered in a school environment to identify at-risk subjects at a very early stage. Early diagnosis would allow appropriate treatment to be initiated, improving the prognosis of these patients.

## Strength and limits

This work is an observational prospective case–control study. The preliminary data showed the existence of linguistic parameters as probable linguistic markers of AN. The analysis of a bigger cohort, still ongoing, is needed to consolidate this assumption.

## What is already known on this subject?

Linguistic deficits have been reported in several neurodegenerative diseases such as dementia. Some studies deal with the linguistic habits of psychopathologies but very few papers have been devoted to linguistic changes in patients with eating disorders.

## What this study adds?

This is the first study on the linguistic profiling of AN-affected individuals in Italy. The use of an automatic system analyzing patients’ language use in written productions may offer significant support for the identification of both overtly pathological and sub-clinical conditions and contribution to early treatment.

## Supplementary Information

Below is the link to the electronic supplementary material.

Supplementary file1 (docx 59 kb)

## Data Availability

The data that support the findings of this study are not publicly available due to restrictions imposed by the Italian legislation. They are available from the corresponding author, upon reasonable request.

## References

[1] Crystal D (1981). Clinical Linguistics.

[2] Marini A, Carlomagno S (2004). Analisi del discorso e patologia del linguaggio.

[3] Adornetti I (2018). Patologie del linguaggio e della comunicazione.

[4] Gagliardi G (2019). Linguistica per le professioni sanitarie.

[5] Konopasky A, Durning SJ (2020). The linguistic effects of context specificity: exploring affect, cognitive processing, and agency in physicians' think-aloud reflections. Diagnosis (Berlin, Germany)..

[6] Boschi V, Catricalà E (2017). Connected speech in neurodegenerative language disorders: a review. Front Psychol.

[7] Beltrami D, Gagliardi G, Rossini Favretti R, Ghidoni E, Tamburini F, Calzà L (2018). Speech analysis by natural language processing techniques: a possible tool for very early detection of cognitive decline?. Front Aging Neurosci.

[8] Gagnon M, Barrette J, Macoir J (2018). Language disorders in huntington disease: a systematic literature review. Cogn Behav Neurol.

[9] Catricalà E, Boschi V (2019). The language profile of progressive supranuclear palsy. Cortex.

[10] Altmann LJ, Troche MS (2011). High-level language production in Parkinson's disease: a review. Parkinsons Dis.

[11] Montemurro S, Mondini S, Signorini M, Marchetto A, Bambini V, Arcara G (2019). Pragmatic language disorder in parkinson's disease and the potential effect of cognitive reserve. Front Psychol.

[12] Dovetto FM, Mariottini L (2015). Uso delle parole nella schizofrenia. Identità e discorsi.

[13] Bambini V, Arcara G (2016). The communicative impairment as a core feature of schizophrenia: Frequency of pragmatic deficit, cognitive substrates, and relation with quality of life. Compr Psychiatry.

[14] de Boer JN, van Hoogdalem M (2020). Language in schizophrenia: relation with diagnosis, symptomatology and white matter tracts. NPJ Schizophr..

[15] Arntz A, Hawke L, Bamelis L, Spinhovend P, Molendijk M (2012). Changes in natural language use as an indicator of psychotherapeutic change in personality disorders. Behav Res Ther.

[16] Ramirez-Esparza N, Chung C, Kacewicz E, and Pennebaker J (2008). The psychology of word use in depression forums in English and in Spanish: Testing two text analytic approaches. In E. Adar, et al., (ed) Second International Conference on We-blogs and Social Media, ICWSM 2008, pp 102–110. AAAI Press, Menlo Park, CA

[17] Brockmeyer T, Zimmermann J (2015). Me, myself, and I: self-referent word use as an indicator of self-focused attention in relation to depression and anxiety. Frontiers in Psychology.

[18] Bernard J, Baddeley J, Rodriguez B, Burke P (2016). Depression, language, and affect: an examination of the influence of baseline depression and affect induction on language. J Lang Soc Psychol.

[19] Edwards T, Holtzman N (2017). A meta-analysis of correlations between depression and first person singular pronoun use. J Res Pers.

[20] Zimmermann J, Brockmeyer T, Hunn M, Schauenburg H, Wolf M (2017). First-person pronoun use in spoken language as a predictor of future depressive symptoms: preliminary evidence from a clinical sample of depressed patients. Clin Psychol Psychother.

[21] Al-Mosaiwi M, Johnstone T (2018). In an absolute state: elevated use of absolutist words is a marker specific to anxiety, depression, and suicidal ideation. Clinical Psychological Science.

[22] Smirnova D, Cumming P, Sloeva E, Kuvshinova N, Romanov D, Nosachev G (2018). Language patterns discriminate mild depression from normal sadnessand euthymic state. Front Psych.

[23] Lyons E, Mehlb M, Pennebaker J (2006). Pro-anorexics and recovering anorexics differ in their linguistic internet self-presentation. Journal of Psychosomatic Research.

[24] Espeset E, Gulliksen K, Nordbø RH, Skårderud F, Holte A (2012). Fluctuations of body images in anorexia nervosa: patients’ perception of contextual triggers. Clin Psychol Psychother.

[25] Skårderud F (2007). Eating one’s words, part I: ‘concretised metaphors’ and reflective function in Anorexia Nervosa—an interview study. Eur Eat Disord Rev.

[26] Skårderud F (2007). Eating one’s words, part II: the embodied mind and reflective function in Anorexia Nervosa—theory. Eur Eat Disord Rev.

[27] Wolf M, Theis F, Kordy H (2013). Language use in eating disorder blogs: psychological implications of social online activity. J Lang Soc Psychol.

[28] Brockmeyer T, Holtforth MG, Bents H, Herzog W, Friederich H (2013). Lower body weight is associated with less negative emotions in sad autobiographical memories of patients with Anorexia Nervosa. Psychiatry Res.

[29] Spinczyk D, Nabrdalik K, Rojewska K (2018). Computer aided sentiment analysis of anorexia nervosa patients’ vocabulary. Biomed Eng Online.

[30] Zipfel S, Giel KE, Bulik CM, Hay P, Schmidt U (2015). Anorexia nervosa: aetiology, assessment, and treatment. Lancet Psychiatry.

[31] Herpertz-Dahlmann B, Dahmen B (2019). Children in need-diagnostics, epidemiology, treatment and outcome of early onset Anorexia nervosa. Nutrients.

[32] Treasure J, Zipfel S, Micali N (2015). Anorexia nervosa. Nat Rev Dis Primers.

[33] Gigantesco A, Masocco M, Picardi A, Lega I, Conti S, Vichi M (2010). Hospitalization for anorexia nervosa in Italy. Riv Psichiatr.

[34] Holliday J, Tchanturia K, Landau S, Collier D, Treasure J (2005). Is impaired set-shifting an endophenotype of anorexia nervosa. J Psychiatry.

[35] Treasure JL (2007). Getting beneath the phenotype of anorexia nervosa: the search for viable endophenotypes and genotypes. Can J Psychiatry.

[36] Wade TD (2008). Shared temperament risk factors for anorexia nervosa: a twin study. Psychosom Med.

[37] Herzog DB, Nussbaum KM, Marmor AK (1996). Comorbidity and outcome in eating disorders. Psychiatr Clin North Am.

[38] Becker AE, Grinspoon SK, Klibanski A, Herzog DB (1999). Eating disorders. N Engl J Med.

[39] Vocks S, Busch M, Grönemeyer D, Schulte D, Herpertz S, e Suchan B,  (2010). Neural correlates of viewing photographs of one’s own body and another woman’s body in anorexia and bulimia nervosa: an fMRI study. J Psychiatry Neurosci.

[40] Konstantakopoulos G, Varsou E (2012). Delusionality of body image beliefs in eating disorders. Psychiatry Res.

[41] Dakanalis A, Clerici M, Carrà G, Riva G (2016). Dysfunctional bodily experiences in anorexia nervosa: where are we?. Eat Weight Disord.

[42] Garner DM (2004). The Eating Disorder Inventory-3: Professional manual.

[43] Goodglass H, Kaplan E, and Barresi B (2001) The Boston Diagnostic Aphasia Examination (BDAE)

[44] Agosti A and Rellini A (2007) The Italian LIWC dictionary. Technical Report 1, Austin, TX

[45] Tausczik Y, Pennebaker J (2010). The psychological meaning of words: LIWC and computerized text analysis methods. J Lang Soc Psychol.

[46] Chung C, Pennebaker J, Fiedler K (2007). The psychological functions of function words. Social Communication.

[47] Rubin D, Greene K (1992). Gender-typical style in written language. Res Teach Engl.

[48] Gavin J, Rodham K, Poyer H (2008). The presentation of “pro-anorexia” in online group interactions. Qual Health Res.

[49] Boto J, Gkinis G, Roche A (2017). Evaluating anorexia-related brain atrophy using MP2RAGE-based morphometry. Eur Radiol.

[50] Enckell H (2002). Metaphor and the psychodynamic functions of the mind. Ph.D. thesis, Kuopion Yliopisto, Kuopio, Finland

[51] Honore A (1979). Some simple measures of richness of vocabulary. Assoc Literary Linguist Comput Bull.

[52] Seitz J, Walter M, Mainz V (2015). Brain volume reduction predicts weight development in adolescent patients with anorexia nervosa. J Psychiatr Res.

