# Supplementary material for: Linguistic feature of anorexia nervosa: a prospective case–control pilot study
Source: Eat Weight Disord. 2021 Jul 26;27(4):1367–75. doi: 10.1007/s40519-021-01273-7 (PMC8311399; doi:10.1007/s40519-021-01273-7)
Supplement: Supplementary file 1 — Supplementary file1 (docx 59 kb) [file 40519_2021_1273_MOESM1_ESM.docx]

| **RESULTS OF LEXICAL AND SYNTACTIC FEATURES EXTRACTION (mean ± standard deviation)** | | | | | | | | |
| --- | --- | --- | --- | --- | --- | --- | --- | --- |
| **Feature** | **task 1 -PERSONAL-** | | **task 2 -NEUTRAL-** | | **task 3 -FIGURE-** | | **overall** | |
|  | **AN** | **CG** | **AN** | **CG** | **AN** | **CG** | **AN** | **CG** |
| LEX_NW | 98.63 ± 42.94 | 105.5 ± 35.05 | 61.53 ± 40.98 | 68.56 ± 31.55 | 81.50 ± 40.02 | 77.15 ± 24.13 | 80.22 ± 43.16 | 83.74 ± 34.18 |
| LEX_ContDens | 1.32±0.19 | 1.37±0.17 | 1.20± 0.23 | 1.14±0.19 | 1.17±0.15 | 1.07±0.14 | 1.22±0.20 | 1.19±0.21 |
| LEX_PoS_*  ADJ  ADV  ART  CONJ  DATE  INTERJ  NOUN  NUM  PHRAS  PREDET  PREP  PRON  VERB | 0.13±0.02  0.10±0.04  0.06±0.03  0.08±0.03  0.00±0.00  0.00±0.00  0.14±0.03  0.01±0.01  0.00±0.00  0.00±0.00  0.08±0.03  0.07±0.03  0.20±0.04 | 0.11±0.03  0.12±0.04  0.07±0.02  0.09±0.02  0.00±0.00  0.00±0.00  0.13±0.03  0.01±0.01  0.00±0.00  0.00±0.00  0.07±0.03  0.07±0.02  0.21±0.03 | 0.06±0.03  0.09±0.05  0.06±0.03  0.08±0.04  0.00±0.00  0.00±0.00  0.18±0.06  0.00±0.01  0.00±0.00  0.00±0.00  0.13±0.06  0.06±0.05  0.20±0.06 | 0.05±0.03  0.11±0.05  0.05±0.02  0.09±0.03  0.00±0.00  0.00±0.00  0.17±0.04  0.00±0.01  0.00±0.00  0.00±0.00  0.14±0.04  0.07±0.04  0.20±0.03 | 0.06±0.03  0.07±0.04  0.11±0.03  0.06±0.03  0.00±0.01  0.00±0.00  0.19±0.03  0.01±0.01  0.00±0.00  0.00±0.00  0.11±0.04  0.07±0.04  0.21±0.04 | 0.04±0.03  0.06±0.04  0.12±0.02  0.07±0.02  0.00±0.00  0.00±0.00  0.20±0.03  0.02±0.01  0.00±0.00  0.00±0.01  0.11±0.03  0.08±0.03  0.22±0.04 | 0.08±0.04  0.09±0.05  0.08±0.04  0.07±0.03  0.00±0.01  0.00±0.00  0.17±0.05  0.01±0.01  0.00±0.00  0.00±0.00  0.12±0.05  0.07±0.04  0.20±0.05 | 0.07±0.04  0.10±0.05  0.08±0.04  0.08±0.03  0.00±0.00  0.00±0.00  0.17±0.04  0.01±0.01  0.00±0.00  0.00±0.00  0.11±0.05  0.07±0.03  0.21±0.03 |
| LEX_RefRReal | 0.72±0.23 | 0.67±0.17 | 1.04±0.71 | 0.92±0.33 | 0.99±0.27 | 0.96±0.24 | 0.93±0.47 | 0.85±0.28 |
| LEX_PDEIXIS  LEX_SDEIXIS  LEX_TDEIXIS | 0.04±0.03  0.00±0.00  0.01±0.01 | 0.04±0.02  0.00±0.00  0.01±0.01 | 0.03±0.03  0.00±0.00  0.01±0.01 | 0.04±0.03  0.00±0.00  0.01±0.01 | 0.03±0.01  0.00±0.00  0.00±0.01 | 0.03±0.02  0.01±0.01  0.00±0.00 | 0.03±0.02  0.00±0.00  0.01±0.01 | 0.04±0.02  0.00±0.01  0.00±0.01 |
| LEX_RPRO  LEX_NEGADV | 0.01±0.01  0.02±0.01 | 0.01±0.01  0.02±0.01 | 0.01±0.01  0.00±0.01 | 0.01±0.01  0.01±0.01 | 0.02±0.02  0.01±0.02 | 0.02±0.02  0.01±0.01 | 0.01±0.02  0.01±0.01 | 0.01±0.02  0.01±0.01 |
| LEX_TTR  LEX_BrunetW  LEX_HonoreR | 0.69±0.06  9.63±1.01  2408.7±659.1 | 0.69±0.06  9.85±0.68  2197.3± 480.0 | 0.79±0.07  8.38±1.23  2325.4±679.8 | 0.75±0.08  8.90±0.98  2172.6±703.0 | 0.76±0.07  9.15±0.78  2326.0±765.8 | 0.73±0.07  9.25±0.76  2026.1±747.5 | 0.75±0.08  9.04±1.12  2351.8±692.2 | 0.73±0.07  9.33±0.90  2131.9±652.0 |
| LEX_ACTVRB | 0.04±0.03 | 0.03±0.02 | 0.05±0.02 | 0.05±0.03 | 0.07±0.03 | 0.52±0.03 | 0.05±0.03 | 0.05±0.03 |
| LEX_DM_F | 3.08±0.57 | 3.11±0.55 | 2.12±0.76 | 2.10±0.67 | 1.54±0.98 | 1.24±0.55 | 2.22±1.01 | 2.15±0.96 |
| LEX_IDEAD | 0.59±0.05 | 0.61±0.04 | 0.56±0.07 | 0.59±0.06 | 0.54±0.05 | 0.52±0.04 | 0.56±0.06 | 0.57±0.06 |
| SYN_NPLENM  SYN_NPLENSD | 2.39±0.70  1.95±0.80 | 2.46±0.58  1.84±0.61 | 1.76±0.68  1.23±1.05 | 1.97±0.59  1.49±0.68 | 2.10±0.59  1.98±0.65 | 1.69±0.49  1.51±0.91 | 2.08±0.69  1.72±0.90 | 2.04±0.63  1.61±0.76 |
| SYN_GRAPHDISTM  SYN_GRAPHDISTD | 1.34±0.29  0.37±0.15 | 1.46±0.20  0.44±0.16 | 1.59±0.35  0.26±0.17 | 1.67±0.21  0.34±0.24 | 1.66±0.43  0.45±0.25 | 1.72±0.29  0.46±0.32 | 1.54±0.38  0.36±0.21 | 1.62±0.26  0.42±0.25 |
| SYN_ISynCompl | 0.34±0.03 | 0.36±0.04 | 0.41±0.05 | 0.42±0.05 | 0.39±0.04 | 0.41±0.06 | 0.38±0.05 | 0.40±0.06 |
| SYN_MAXDEPTHM  SYN_MAXDEPTHD | 7.60±2.75  2.48±1.44 | 7.40±1.37  2.52±1.16 | 7.16±1.53  1.87±1.42 | 8.50±3.29  2.07±1.90 | 8.73±2.64  2.56±1.55 | 8.87±3.86  3.04±1.73 | 7.85±2.42  2.30±1.48 | 8.25±3.07  2.54±1.66 |
| SYN_SLENM SYN_SLENSD | 17.44±6.92  5.64±2.89 | 19.27±4.93  8.73±4.59 | 19.54±5.30  5.58±4.60 | 25.84±11.81  7.60±7.25 | 24.50±10.26  8.37±5.26 | 26.38±11.05  9.41±6.42 | 20.63±8.26  6.58±4.52 | 23.83±10.20  8.58±6.17 |

| **RESULTS OF LIWC FEATURES EXTRACTION (mean ± standard deviation)** | | | | | | | | |
| --- | --- | --- | --- | --- | --- | --- | --- | --- |
| **Feature** | **task 1 -PERSONAL-** | | **task 2 -NEUTRAL-** | | **task 3 -FIGURE-** | | **overall** | |
|  | **AN** | **CG** | **AN** | **CG** | **AN** | **CG** | **AN** | **CG** |
| WC | 85.76±36.64 | 92.76±31.20 | 53.71±35.89 | 61.67±29.40 | 72.41±35.17 | 70.03±21.36 | 70.63±37.60 | 74.82±30.40 |
| WPS | 15.68±6.55 | 16.77±3.99 | 16.67±5.04 | 23.17±11.39 | 24.48±9.51 | 24.64±11.43 | 18.94±8.16 | 21.52±10.10 |
| SIXLTR | 26.23±5.31 | 22.91±3.94 | 27.71±7.29 | 24.38±6.10 | 28.18±6.60 | 25.43±4.30 | 27.37±6.38 | 24.24±4.93 |
| DIC | 63.09±5.01 | 65.90±4.95 | 59.77±5.84 | 64.20±7.66 | 59.57±4.54 | 67.16±5.54 | 60.81±5.31 | 65.76±6.22 |
| 1PS | 12.74±2.98 | 14.10±2.94 | 5.09±3.86 | 6.21±5.10 | 1.87±3.22 | 1.53±1.25 | 6.57±5.67 | 7.28±6.25 |
| 1PP | 0.00±0.00 | 0.12±0.35 | 6.40±5.25 | 5.67±3.92 | 0.36±0.73 | 0.10±1.14 | 2.25±4.22 | 2.26±3.39 |
| 2PS | 0.00±0.00 | 0.00±0.00 | 0.00±0.00 | 0.00±0.00 | 0.00±0.00 | 0.00±0.00 | 0.00±0.00 | 0.00±0.00 |
| 2PP | 0.00±0.00 | 0.00±0.00 | 0.00±0.00 | 0.00±0.00 | 0.00±0.00 | 0.00±0.00 | 0.00±0.00 | 0.00±0.00 |
| 3PS | 0.04±0.16 | 0.02±0.13 | 0.00±0.00 | 0.00±0.00 | 0.16±0.45 | 0.19±0.47 | 0.06±0.28 | 0.07±0.29 |
| 3PP | 0.00±0.00 | 0.00±0.00 | 0.00±0.00 | 0.00±0.00 | 0.00±0.00 | 0.00±0.00 | 0.00±0.00 | 0.00±0.00 |
| NEG | 2.44±2.27 | 2.29±1.33 | 0.26±0.74 | 1.23±1.80 | 1.15±1.52 | 1.49±1.41 | 1.28±1.84 | 1.67±1.58 |
| PST | 0.25±0.74 | 0.08±0.35 | 0.58±1.34 | 0.07±0.43 | 0.16±0.54 | 0.24±0.73 | 0.33±0.94 | 0.13±0.53 |
| PRES | 13.46±3.80 | 15.08±2.89 | 10.33±5.58 | 11.19±4.89 | 7.08±3.18 | 9.54±2.49 | 10.29±4.98 | 11.94±4.24 |
| FUT | 0.00±0.00 | 0.04±0.20 | 0.00±0.00 | 0.00±0.00 | 0.14±0.40 | 0.34±0.63 | 0.05±0.24 | 0.12±0.41 |
| GER | 0.05±0.20 | 0.03±0.20 | 0.35±0.71 | 0.19±0.75 | 1.70±2.41 | 1.98±2.14 | 0.67±1.60 | 0.73±1.57 |
| COND | 0.78±1.08 | 0.31±0.55 | 0.07±0.29 | 0.05±0.21 | 0.28±0.69 | 0.4±0.72 | 0.38±0.80 | 0.25±0.55 |
| PASS | 0.00±0.00 | 0.00±0.00 | 0.00±0.00 | 0.00±0.00 | 0.00±0.00 | 0.00±0.00 | 0.00±0.00 | 0.00±0.00 |
| PP | 0.56±0.90 | 0.4±0.58 | 0.50±0.94 | 0.47±1.01 | 0.95±1.14 | 0.96±1.06 | 0.67±1.00 | 0.61±0.93 |
| TRAN | 0.18±0.40 | 0.35±0.70 | 0.99±1.50 | 0.20±0.73 | 1.55±1.27 | 2.03±1.17 | 0.91±1.27 | 0.86±1.21 |
| AFFP | 8.06±3.06 | 7.92±3.16 | 2.29±2.40 | 3.52±3.01 | 1.75±1.98 | 1.2±2.12 | 4.03±3.80 | 4.21±3.94 |
| +EMO | 4.60±2.72 | 3.86±2.28 | 1.02±1.47 | 0.97±1.47 | 0.48±0.80 | 0.12±0.40 | 2.03±2.58 | 1.65±2.25 |
| -EMO | 1.67±1.27 | 1.71±1.51 | 0.46±1.35 | 0.81±1.34 | 0.97±1.29 | 0.81±1.77 | 1.03±1.37 | 1.11±1.59 |
| ANX | 0.45±0.84 | 0.26±0.52 | 0.00±0.00 | 0.07±0.24 | 0.06±0.26 | 0.10±0.33 | 0.17±0.54 | 0.14±0.39 |
| ANG | 0.70±1.03 | 0.77±1.24 | 0.00±0.00 | 0.06±0.37 | 0.32±0.72 | 0.19±0.69 | 0.34±0.77 | 0.34±0.89 |
| SAD | 0.12±0.36 | 0.42±0.71 | 0.20±0.66 | 0.41±0.95 | 0.26±0.62 | 0.43±0.91 | 0.20±0.56 | 0.42±0.85 |
| COGP | 4.91±2.59 | 4.92±2.95 | 1.32±2.18 | 2.81±2.72 | 3.04±2.10 | 4.44±2.76 | 3.09±2.70 | 4.06±2.93 |
| INS | 1.37±1.28 | 1.62±1.70 | 0.55±1.38 | 0.84±1.39 | 1.05±1.15 | 2.24±2.18 | 0.99±1.29 | 1.56±1.86 |
| CAU | 0.45±0.67 | 0.38±0.75 | 0.00±0.00 | 0.38±0.77 | 0.33±0.63 | 0.34±0.64 | 0.26±0.55 | 0.37±0.71 |
| DISCR | 1.77±1.57 | 2.14±1.65 | 0.35±0.76 | 0.99±1.54 | 0.08±0.95 | 1.10±1.21 | 0.97±1.27 | 1.41±1.55 |
| TENT | 3.08±1.90 | 3.55±2.13 | 3.21±2.05 | 4.33±2.65 | 1.75±1.69 | 1.83±1.79 | 2.68±1.96 | 3.24±2.44 |
| CERT | 0.97±1.12 | 1.49±1.54 | 0.51±1.11 | 0.88±1.36 | 0.46±1.13 | 0.38±0.68 | 0.65±1.12 | 0.91±1.32 |
| INH | 0.44±0.69 | 0.30±0.48 | 0.10±0.41 | 0.08±0.36 | 0.03±0.13 | 0.15±0.44 | 0.19±0.49 | 0.18±0.44 |
| INCL | 0.78±1.06 | 1.07±1.17 | 1.20±1.48 | 0.78±1.28 | 0.50±0.84 | 1.02±1.17 | 0.83±1.17 | 0.96±1.20 |
| EXCL | 3.83±2.17 | 4.80±2.40 | 4.17±3.00 | 5.32±3.56 | 3.70±2.34 | 4.88±2.17 | 3.90±2.49 | 5.00±2.76 |
| PERCP | 3.12±2.04 | 2.71±1.52 | 1.55±1.95 | 2.80±2.09 | 0.85±0.90 | 1.39±1.48 | 1.84±1.93 | 2.30±1.82 |
| SEE | 1.79±1.51 | 1.14±0.96 | 0.63±1.74 | 0.55±0.95 | 0.52±0.78 | 0.77±1.00 | 0.98±1.49 | 0.82±0.99 |
| HEAR | 0.73±1.18 | 1.13±1.18 | 0.74±1.10 | 1.83±1.61 | 0.00±0.00 | 0.00±0.00 | 0.49±0.98 | 0.99±1.37 |
| FEEL | 0.14±0.31 | 0.20±0.62 | 0.00±0.00 | 0.09±0.39 | 0.09±0.28 | 0.27±0.55 | 0.08±0.24 | 0.19±0.53 |
| BODY | 4.32±1.87 | 3.98±2.38 | 0.30±0.70 | 1.15±1.57 | 1.88±1.27 | 2.55±1.51 | 2.17±2.14 | 2.56±2.18 |
| HLT | 0.76±0.68 | 0.52±0.56 | 0.00±0.00 | 0.04±0.26 | 0.03±0.13 | 0.19± 0.57 | 0.26±0.52 | 0.25±0.52 |
| ING | 0.79±1.31 | 0.36±0.91 | 0.47±0.91 | 0.61±1.27 | 2.12±1.46 | 2.46±1.08 | 1.13±1.42 | 1.15±1.44 |
| WORK | 0.13±0.37 | 0.15±0.36 | 0.30±0.96 | 0.04±0.21 | 0.00±0.00 | 0.00±0.00 | 0.14±0.60 | 0.06±0.25 |
| SCHOOL | 0.36±0.76 | 0.32±1.04 | 0.85±2.05 | 0.23±0.64 | 0.00±0.00 | 0.00±0.00 | 0.40±1.28 | 0.18±0.71 |
| DEATH | 0.00±0.00 | 0.00±0.00 | 0.00±0.00 | 0.00±0.00 | 0.00±0.00 | 0.00±0.00 | 0.00±0.00 | 0.00±0.00 |
| ACH | 0.38±0.79 | 0.43±0.79 | 0.13±0.54 | 0.22±0.57 | 0.39±0.76 | 0.45±0.70 | 0.30±0.70 | 0.37±0.69 |
| LEIS | 1.50±1.28 | 0.96±1.49 | 2.36±1.82 | 3.10±1.96 | 1.60±1.93 | 1.18±1.00 | 1.82±1.72 | 1.75±1.80 |
| HOME | 0.70±1.01 | 0.30±0.62 | 0.77±1.17 | 0.89±1.01 | 1.52±1.9 | 1.18±1.00 | 1.00±1.44 | 0.79±0.96 |
| SPORT | 0.00±0.00 | 0.10±0.40 | 0.00±0.00 | 0.35±1.23 | 0.00±0.00 | 0.00±0.00 | 0.00±0.00 | 0.15±0.76 |
| FAM | 0.33±0.54 | 0.17±0.41 | 0.00±0.00 | 0.06±0.32 | 3.30±2.08 | 3.50±1.88 | 1.21±1.93 | 1.24±1.95 |
| FR | 0.80±1.10 | 0.77±0.86 | 2.10±2.10 | 2.01±1.80 | 0.03±0.13 | 0.00±0.00 | 0.98±1.60 | 0.92±1.41 |
| HUM | 2.08±1.60 | 2.07±1.37 | 0.77±1.75 | 0.18±0.55 | 3.64±2.18 | 3.88±2.34 | 2.17±2.18 | 2.04±2.19 |
| SOC | 4.95±2.90 | 4.75±2.33 | 8.69±5.41 | 8.60±5.09 | 8.09±2.32 | 9.01±2.63 | 7.24±4.06 | 7.45±4.03 |
